# Integration of Multiple Genomic Data Sources in a Bayesian Cox Model for Variable Selection and Prediction

**DOI:** 10.1155/2017/7340565

**Published:** 2017-07-30

**Authors:** Tabea Treppmann, Katja Ickstadt, Manuela Zucknick

**Affiliations:** ^1^EXCO, Penzberg, Germany; ^2^Department of Statistics, TU Dortmund University, Dortmund, Germany; ^3^Oslo Centre for Biostatistics and Epidemiology, Department of Biostatistics, Institute of Basic Medical Sciences, University of Oslo, Oslo, Norway

## Abstract

Bayesian variable selection becomes more and more important in statistical analyses, in particular when performing variable selection in high dimensions. For survival time models and in the presence of genomic data, the state of the art is still quite unexploited. One of the more recent approaches suggests a Bayesian semiparametric proportional hazards model for right censored time-to-event data. We extend this model to directly include variable selection, based on a stochastic search procedure within a Markov chain Monte Carlo sampler for inference. This equips us with an intuitive and flexible approach and provides a way for integrating additional data sources and further extensions. We make use of the possibility of implementing parallel tempering to help improve the mixing of the Markov chains. In our examples, we use this Bayesian approach to integrate copy number variation data into a gene-expression-based survival prediction model. This is achieved by formulating an informed prior based on copy number variation. We perform a simulation study to investigate the model's behavior and prediction performance in different situations before applying it to a dataset of glioblastoma patients and evaluating the biological relevance of the findings.

## 1. Introduction

In cancer research, we often deal with time-to-event endpoints, and the more advances in technology enable the systematic collection of different genome-wide data, the more interest arises in integrative statistical analyses, that is, using more than one information source to obtain a more comprehensive understanding of the biology of diseases and improve the performance of risk prediction models.

Recently, a lot of research has been done in the following three areas:

1. Cox proportional hazards models for survival (or time-to-event) data in high dimensions
2. For variable selection in high-dimensional problems
3. For integrative analyses of several data sources

The novelty of our approach is the combination of recent advances in these three areas in one Bayesian model as outlined below.

(1) To model* survival data*, Cox (1972) [1] developed the semiparametric proportional hazards regression model for taking into account the relation between covariates and the hazard function. The Cox model has been widely used and analyzed in low-dimensional settings for this purpose; see, for example, Harrell Jr. (2001) [2], Klein et al. (2013) [3], or Ibrahim et al. (2005) [4]. In biological applications with genomic data, we are, however, often in a high-dimensional setting, that is, having more variables than subjects. Therefore, we are in need of a high-dimensional survival time model. One recent approach in this context was suggested by Lee et al. (2011) [5], who use a Bayesian version of the Cox model for right censored survival data, where high dimensions are handled by regularization of the regression coefficient vector imposed by Laplace priors. This corresponds to the lasso penalty; see Tibshirani (1997) [6] or Park and Casella (2008) [7], which shrinks regression coefficients towards zero and thus allows parameter inference in problems where the number of variables *p* is larger than number of subjects *n*. Since the automatic variable selection property of lasso is lost in fully Bayesian inference, Lee et al. (2011) [5] adopted a post hoc approach to identify the most important variables by thresholding based on the Bayesian Information Criterion.

(2) Since* variable selection* is a core question in many statistical applications, it has been subject to a lot of research, and many approaches exist, especially for linear models. In low-dimensional settings and for frequentist inference, the most common procedures are best subset selection or backward or forward selection (Harrell Jr. (2001) [2], Hocking, (1976) [8]). There are 2^*p*^ different models to evaluate for best subset selection which becomes infeasible in higher dimensions (*p* > 30). In high dimensions, classical backward selection cannot be applied since the full model is not identified, and both backward and forward selection will typically only explore a very small proportion of all possible models. In addition, all of these approaches do not incorporate shrinkage in the estimation procedure. Bayesian approaches offer a good alternative to stochastically search over the whole parameter space, implicitly taking into account the model uncertainty; see Held et al. (2016) [9] for a recent evaluation study in the context of Cox regression models. One appealing approach often used in regression analyses is the stochastic search variable selection (SSVS) of George and McCulloch (1993) [10], a flexible and intuitive method which makes use of data augmentation for the selection task and incorporates shrinkage.

(3) For biological information on a molecular level, many* different data sources* exist nowadays, and they often provide shared information, for example, the amount of expressed genes being transcribed to different proteins results in different functions of the cells or the body. If unexpected or unusual changes in the expression levels occur, the functionality of the cells can be disturbed. Cancer is often caused by changes in the DNA, for example, single-base mutations or copy number changes in larger genomic regions, which in turn will have an effect on gene expression. Therefore, including such data sources jointly into the analyses can lead to more accurate results. Bayesian approaches offer a handy pipeline to do so.

*In our approach* we combine the three mentioned tasks in one model: variable selection in a high-dimensional survival time model based on an integrative analysis. In particular, we integrate copy number variation (CNV) data with gene expression data, aiming to jointly use their respective advantages to achieve sparse and well interpretable models and good prediction performance. We combine the variable selection procedure of George and McCulloch (1993) [10] with the Cox proportional hazards model of Lee et al. (2011) [5] and use CNV data for the construction of an informed prior. We investigate the use of parallel tempering methods to improve the mixture of the Markov chains and to circumvent the manual tuning of hyperprior parameters.

In the following, we describe the details of the model, including the technical details, the sampler with extensions, and diagnostics, in Section 2. Afterwards, we describe the synthetic data as well as the real dataset on glioblastoma; we state the prior settings needed and chosen for the simulation study as well as for the real data analysis. Before drawing conclusions in Section 4, we describe the most important findings for the application to synthetic and real data, including findings regarding the extracted genes for glioblastoma patients, and discuss the results in Section 3.

## 2. Materials and Methods

### 2.1. Model and MCMC Sampling Procedure

Based on the general semiparametric proportional hazards model *λ*(*t*∣*x*) = *h*_0_(*t*) × exp(*x*′*β*) introduced by Cox (1972) [1], Lee et al. (2011) [5] developed a Bayesian version for right censored survival time data in high dimensions (*p* ≫ *n*), with *p* being the number of variables, *n* the number of subjects, *t* the survival time of a person with covariable vector *x* = (*x*_1_,…,*x*_*p*_)′, *β* = (*β*_1_,…,*β*_*p*_)′ the vector of regression parameters, and *h*_0_(*t*) the unspecified arbitrary baseline hazard function. Lee et al. (2011) constructed a grouped likelihood for their model with a finite partitioning of the time axis, 0 < *s*_0_ < *s*_1_ < *s*_2_ < ⋯<*s*_*J*_ with *s*_*J*_ > *t*_*r*_, ∀*r* = 1,…, *n*, in this case choosing the breaks as the points at which at least one event occurred and defining the last interval so that the last event lies in the middle of it, leading to the grouped data likelihood introduced by Burridge (1981) [11]

(1) LD ∣ β,h∝∏j=1Jexp−hj×∑ι∈Rj−Djexpxι′β×∏ξ∈Dj1−exp−hj×expxξ′βhj~Gα0j−α0j−1,c0,j=1,…,J.

Here, **D** = {(*x*, *ℛ*_*j*_, *𝒟*_*j*_) : *j* = 1,…, *J*} denotes the observed data, where *ℛ*_*j*_ and *𝒟*_*j*_ are the risk sets and the event sets corresponding to the *j*th interval. *G*(·) describes a Gamma distribution with shape *α*_0_*j*__ − *α*_0_*j*−1__ and scale *c*_0_, where *α*_0_*j*__ = *c*_0_ × *H*^*∗*^(*s*_*j*_),  *j* = 1,…, *J*, and *H*^*∗*^(*t*) is a monotonously increasing function with *H*^*∗*^(0) = 0. *H*^*∗*^(0) represents an initial estimate for the cumulative baseline hazard function *H*_0_(*t*). The constant *c*_0_ > 0 specifies how strong the believe in the initial estimate for this cumulative baseline hazard function is. Mostly, a known parametric function for *H*^*∗*^(*t*) is used, for example, the Weibull distribution, which then leads to the following form:

(2) $$\begin{array}{c} H^{*}\left(\;t\;\right)=\eta_{0}\times t^{\kappa_{0}}. \end{array}$$

The hyperparameters (*η*_0_, *κ*_0_) have to be carefully chosen, though, to avoid convergence problems within the MCMC sampling [5].

The implicit shrinkage of the model and the variable selection will be done through the stochastic search variable selection procedure of George and McCulloch (1993) [10]. Assuming equal variances for the regression coefficients of variables which are included in the model, the prior distribution for *β*_*i*_ conditioned on *γ*_*i*_, *i* = 1,…, *p* is as follows:

(3) $$\begin{array}{c} \beta_{i}\;|\;\gamma_{i}\sim \left(\;1-\gamma_{i}\;\right)\times N\left(\;0,\tau^{2}\;\right)+\gamma_{i}\times N\left(\;0,c_{b}^{2}\times \tau^{2}\;\right), \end{array}$$

where the variance parameter *τ*^2^ > 0 is small, *c*_*b*_^2^ > 1, and *γ* represents an indicator vector, analogous to the concept of data augmentation (Tanner and Wong, 1987 [12]), giving the state of the respective variable of being in the model or not.

As in Lee et al. (2011) [5], we compared three possible samplers to update the full conditional distribution *P*(*β*_*i*_∣*β*_−*i*_, *γ*, *h*, **D**), (*β*_−*i*_ = (*β*_1_,…,*β*_*i*−1_, *β*_*i*+1_,…,*β*_*p*_)′ and *i* = 1,…, *p*): the Adaptive Rejection Sampling algorithm proposed by Gilks (1992) [13], as well as the Adaptive Rejection Metropolis Sampler from Gilks et al. (1995) [14] and the special random walk Metropolis-Hastings (RW-MH) method with adaptive jumping rules proposed by Lee et al. (2011) [5]. We also found the adaptive random walk Metropolis-Hastings sampler to perform best in our applications, which are high-dimensional with more variables than samples and *p* > 100. We therefore only report the results for the adaptive RW-MH sampler.

*γ*_*i*_ are assumed to be independent Bernoulli (*π*_*i*_) a priori; that is, *P*(*γ*_*i*_ = 1) = *π*_*i*_ and *P*(*γ*_*i*_ = 0) = 1 − *π*_*i*_. The conditional distributions *P*(*γ*_*i*_^*it*^ = 1∣*β*^*it*^, *σ*^*it*^, *γ*_−*i*_^*it*^) with *γ*_−*i*_^*it*^ = (*γ*_1_^*it*^,…,*γ*_*i*−1_^*it*^, *γ*_*i*+1_^*it*^,…,*γ*_*p*_^*it*^)′ for the MCMC sampler are determined by

(4) Pγiit=1 ∣ βit,σit,γ−iit=Pγiit=1 ∣ βiit=aa+b,i=1,…,p,

where

(5) a=fβiit ∣ γiit=1×πi,b=fβiit ∣ γiit=0×1−πi,

with *f*(·∣*γ*_*i*_^*it*^ = 1) being the density of the normal distribution *N*(0, *c*_*b*_^2^ × *τ*^2^) and *f*(·∣*γ*_*i*_^*it*^ = 0) corresponding to *N*(0, *τ*^2^).

According to Ibrahim et al. (2005) [4], the full conditional distribution *P*(*h*_*j*_∣*h*_−*j*_, *β*, *γ*, **D**), with *h*_−*i*_ = (*h*_1_,…,*h*_*j*−1_, *h*_*j*+1_,…,*h*_*J*_)′, can be well approximated by a gamma distribution

(6) hj ∣ h−j,β,γ,D~approx.Gα0j−α0j−1+dj,c0+∑ι∈R−Dexp⁡xι′β,

*j* = 1,…, *J*, where *d*_*j*_ represents the number of censorings in interval *I*_*j*_.

Finally, we use a Gibbs sampler to update *β*, *γ*, and *h* iteratively according to the full conditional distributions described above.

### 2.2. Extension of MCMC Sampling Procedure

For multimodal posterior distributions, some problems may occur during the MCMC sampling, because the areas in the model space with higher posterior probability might be separated by a low-probability region, which the MCMC sampler might not manage to overcome. Therefore, there is a risk that important values cannot be sampled, because the MCMC sampler never visits the relevant region in the model space. Parallel tempering [15, 16] can alleviate this problem. Even in unimodal situations, parallel tempering can help by broadening the area of the sampling. This is done through the parallel generation of *v* + 1 different MCMC chains with their own stationary distributions, where at regular intervals (after a predetermined number of MCMC iterations) a swapping of states (i.e., of the current values of all parameters in the model) of two neighboring chains is proposed. The distributions of all chains have the same basic form as the original, but are more flat. This is achieved by raising the original density function to the power *𝒯*^−1^ (*𝒯* ≥ 1) with values between 0 and 1, with 0 (for *𝒯* → *∞*) corresponding to a complete flattening of the distribution and 1 corresponding to the desired target. This can improve the sampling performance in two ways: (a) the flattened probability distribution covers more of the parameter space with sufficiently large probability to be reached by the sampler in a given number of iterations, and (b) the “hills” and “valleys” of a multimodal probability density will be less steep, thus reducing the likelihood that the sampler might get stuck in local optima (which in turn will improve its mixing performance). For historical reasons, the parameter *𝒯* is usually referred to as a* temperature parameter*.

At regular intervals (in our applications after every tenth MCMC iteration), two neighboring chains are selected randomly, and the Metropolis-Hastings acceptance probability is calculated based on the target distributions and the current states of the chains to determine whether a swap of the states between these two chains is accepted.

Let *f*(*θ*_ch_1__) and *g*(*θ*_ch_2__) be the respective target distributions of the selected chains with current parameter states *θ*_ch_1__ and *θ*_ch_2__. The acceptance probability of swapping states is given by min{1, *α*} with

(7) $$\begin{array}{c} \alpha =\frac{f\left(\theta_{{ch}_{2}}\right)\times g\left(\theta_{{ch}_{1}}\right)}{f\left(\theta_{{ch}_{1}}\right)\times g\left(\theta_{{ch}_{2}}\right)}. \end{array}$$

Within the Metropolis update, this will be compared with a uniform random variable *U* in the interval [0,1], where *U* < min{1, *α*} means that the swap will be accepted. The probability of a chain to swap to another state therefore only depends on the current states of the compared chains [17].

In this manuscript, we use log-linear temperature scales *𝒯*^ch^, (ch = 0,…, 5). The original, untempered chain is hence given by ch = 0. The distributions of the tempered versions are determined so that the standard deviation of the normal mixture prior of *β*∣*γ* (equation (3)) will be broadened, which is achieved by multiplying the parameter *τ* in the prior with *𝒯*^ch^ (ch = 0,…, 5).

It is recommended to choose the temperatures so that the acceptance rate lies between 20% and 50%, since different studies have shown that rates in this range deliver the most satisfactory results (e.g., [16, 18, 19]).

### 2.3. Prior Settings

For the application of the Bayesian model, several prior specifications are needed. We start with the hyperparameters *η*_0_ and *κ*_0_, which are chosen so that *H*^*∗*^(*t*) in (2) is similar to the Nelson-Aalen estimator of the cumulative hazard function, which is therefore used to provide an initial guess for *H*_0_(*t*). For this we determine the scale parameters for the Weibull distribution from the estimated survival model of the event times of the training data without covariable information. For the update of the cumulated baseline hazard *H*_0_(*t*) within the iterations of the MCMC chains, the hyperparameter *c*_0_, which describes the level of certainty associated with *H*^*∗*^, has to be specified. We follow the suggestion by Lee et al. (2011) [5] to set *c*_0_ = 2. We have previously performed a sensitivity analysis to investigate the influence of the choice of *c*_0_ (Zucknick et al., 2015 [20]), where we found that while there was a notable influence on the posterior estimates of the baseline hazard *h*, the posterior distributions of *β* were nearly unchanged.

The parameters *τ* and *c*_*b*_ of the normal mixture distribution of *β* in (3) conditioned on *γ* in (4), that is, *P*(*β*∣*γ*), will be set to *c*_*b*_ = 20 and *τ* = 0.0375. This implies that we obtain a standard deviation of *c*_*b*_ × *τ* = 0.75 for *P*(*β*_*i*_∣*γ*_*i*_ = 1) and a corresponding 95% probability interval of [−1.96,1.96].

The specifications of the prior probabilities for the selection of the variables are described in Section 2.5, separately for the simulation scenarios and for the glioblastoma data application.

### 2.4. Posterior Estimation and Prediction

We report the posterior distributions of *β* and *γ* in terms of their posterior means and standard deviations. In order to select the most relevant variables, we choose an inclusion criterion in an automated data dependent way, which respects the prior model setup instead of choosing one cutoff for all cases. This is done by first calculating the mean model size *p*_*m*_ (by rounding the average of selected variables per iteration). Then we choose *p*_*m*_ variables with the highest selection probability.

We used the empty model, with *β*_*i*_ = 0 for all *i* = 1,…, *p*, as starting values of the MCMC chains.

The results of the simulation study are based on single MCMC chains with 100,000 iterations each, after removal of 20,000 iterations (“burn-in”). The results for the glioblastoma data application are based on a combined analysis of five Markov chains, each of length 90,000 after removal of 10,000 initial iterations (“burn-in”). For the parallel tempering (only applied to the simulated data), we included four chains with 30,000 iterations each and log-linear temperature scales.

We evaluated the mixing and convergence properties of the Markov chains in several ways. We used graphical evaluations of running means plots of the individual *β* parameters as well as trace plots for summary measures such as the *L*_2_-norm of the *β* vector, the model size, and the log likelihood. Additionally, we calculated the effective sample sizes ([21]) for each *β*_*i*_. The R package coda [22] offers a wide variety of graphics and diagnostic measures to assess both mixing and diagnostic performance of MCMC chains.

We evaluate the prediction accuracy of the models chosen this way by prediction error curves and by computing the integrated Brier score (IBS) [23, 24] and comparing them with the reference approach, which is the Kaplan-Meier estimator without any covariates. The Brier score is a strictly proper scoring rule, since it takes its minimum when the true survival probabilities are used as predictions [24, 25]. It therefore measures both discrimination and calibration, contrary to other common measures of evaluation such as Harrell's *c*-Index (which only measures discrimination) and the calibration slope (for measuring calibration); see, for example, Held et al., 2016 [9].

The implementation of the model and the evaluations were done in the statistical computing environment R [26] and are available upon request from the authors.

### 2.5. Data

#### 2.5.1. Simulated Data

For obtaining simulated data for our survival time model, we generated two different datasets, representing a sparse and nonsparse scenario for the true predictors. For the simulation of the survival data, we used the procedure described in Zucknick et al. (2015) [20] for the high-dimensional case. This setup is based on the approach of Bender et al. (2005) [27] following a Cox-Weibull survival model with known regression coefficients and any nonzero baseline hazard rate, taking into account the general relation between the hazard function and the survival time of the Cox model. We simulated blockwise correlated variables with a pairwise correlation *ρ*^|*i*−*j*|^ between variables *i* and *j*, with *ρ* = 0.5 for the variables within the blocks of size *m* = 100.

In short, we first simulate the hypothetical survival times *T*_*l*_^*∗*^ (*l* = 1,…, *n*) that would be observed without the presence of censoring,

(8) Tl∗~−1η expxl′βlog Ul1/κwith  Ul~U0,1,

and the censoring times *C*_*l*_^*∗*^, which are generated to be uninformative and a mixture of uniform administrative censoring and exponential loss to follow-up. Note that scale and shape parameters *η* and *κ* are chosen such that the survival probabilities at 12 and 36 time units are 0.5 and 0.9, respectively. For more details, we refer to Zucknick et al. (2015) [20].

Then, for each subject  *l* = 1,…, *n* the individual observed time to event or censoring *t*_*l*_ and the corresponding survival status *δ*_*l*_ are defined as

(9) tl=min Tl∗,Cl∗,δl=1,Tl∗≤Cl∗0,Tl∗>Cl∗.

For both scenarios, we generate a training dataset for model fitting and a test dataset to evaluate the prediction performance of the final models. The generated datasets comprise *p* = 500 genomic variables and *n* = 200 subjects. In the* sparse setting,* we have true effects of the prognostic variables of *β* = (0.75, −0.75,0.5, −0.5,0.25, −0.25,0,…, 0), analogous to the setup of Zucknick et al. (2015) [20]. Therefore, the first *k*_true_ = 6 variables are simulated to be related to the response (called “predictors” throughout the manuscript). For the* nonsparse setting* we randomly generated *k*_true_ = 122 variables in the range of (−0.8, −0.2)∪(0.2,0.8) and equally distributed for the negative and positive part. Therefore, in this setting, the first *k*_true_ = 122 variables of the dataset represent the true predictors. See Tables 1 and 2 for an overview of all simulation scenarios.

*Prior Inclusion Probabilities*. To evaluate the impact of prior information we investigate three different scenarios for the simulated data. First, we choose an uninformative selection prior (in short: uninformative prior) as *π* = (*k*/*p*,…,*k*/*p*)′, where *k* is the a priori expected number of predictors being set to *k* = 20 here. With this we can assess the model's behavior if no prior knowledge is present. Second, mimicking the influence of correct prior information we set the prior probability of the true variables to 0.8 and the others to 0.1. Finally, to see what happens if our prior knowledge does not represent the truth, we specify a third prior, setting the prior probabilities of *k*_true_ randomly selected variables of the nonpredictors to 0.8 and the remaining variables, which include the true ones, to 0.1.

#### 2.5.2. Application to a Glioblastoma Study

To evaluate our model in a real application, we used a dataset of* glioblastoma multiforme* (GBM) patients, retrieved from* The Cancer Genome Atlas* (TCGA) database [28]. Glioblastoma is the most common and fast-growing brain tumor in adults. It shows a very poor prognosis with a median overall survival time of less than 15 months after diagnosis and a two-year survival rate of about 30% [29]. Therefore, a more detailed understanding of the molecular behavior of glioblastoma tumors is sorely needed. Recent publications studying the genomic profile of glioblastoma include the original publication from the TCGA network (McLendon et al., 2008 [30]) and the follow-up article by Brennan et al. (2013) [31], as well as Sturm et al. (2012) [32].

We extracted the data from two sources: from the GBM dataset of the TCGA Pancancer dataset https://www.synapse.org/#!Synapse:syn1710678 [33] and from the derivative DREAM challenge TCGA Pancancer Survival Prediction project (https://www.synapse.org/#!Synapse:syn1710282) [34]. Our final dataset comprises 210 subjects, for which we matched the patient survival data and gene expression data (from the DREAM challenge dataset) with their respective CNV data retrieved from the PanCan12 dataset. For the analysis, we selected the *p* = 1,000 genes (selected among all genes located on autosomal chromosomes with available annotation information) with the highest variability in their gene expression values across patients, and we matched the copy number variation data to these genes. These 1,000 genes together make up 30% of the total variation in the dataset. The choice of selecting the genes with the largest variance is based on the assumption that genes which do not vary much between subjects will not be helpful in discriminating between patients with poor and good survival prognosis, respectively.

We randomly split the data with ratio 2 : 1 into a training set with *n* = 140 patients for model fitting and a test set with 70 subjects, which we use for the evaluation of the prediction performance of the final models.

*Prior Inclusion Probabilities.* We choose the uninformative prior as *π* = (*k*/*p*,…,*k*/*p*)′ with *k* = 20. In the informative case, we define the prior inclusion probability *π*_*i*_ (*i* = 1,…, *p*) proportional to the standard deviation *σ*_*i*_^CNV^ of the copy number variation data for the associated genomic region across patients times *k*. The prior for gene variable of index *i* is then defined as *π*_*i*_ = *k* × (*σ*_*i*_^CNV^/∑_*j*=1_^*p*^*σ*_*j*_^CNV^) to obtain again *k* as the a priori expected number of selected variables. The empirical distribution of *σ*_*i*_^CNV^ (*i* = 1,…, *p*) is right-skewed with median 0.36, interquartile range (0.28,0.45), and range (0.16,1.45).

## 3. Results and Discussion

### 3.1. Simulation Study

In the simulation study, we use the synthetic data generated as described in Section 2.5.1.

#### 3.1.1. Sparse Setting

First, we look at the sparse scenario where we generated *k*_true_ = 6 true predictors, which correspond to the first *k* variables in our setting. For all three prior settings, we observe that variables with an absolute effect of at least 0.5 will generally be selected by the model (Table 1), though the posterior estimates generally show an overestimation of the true values.

**Figure 1:**
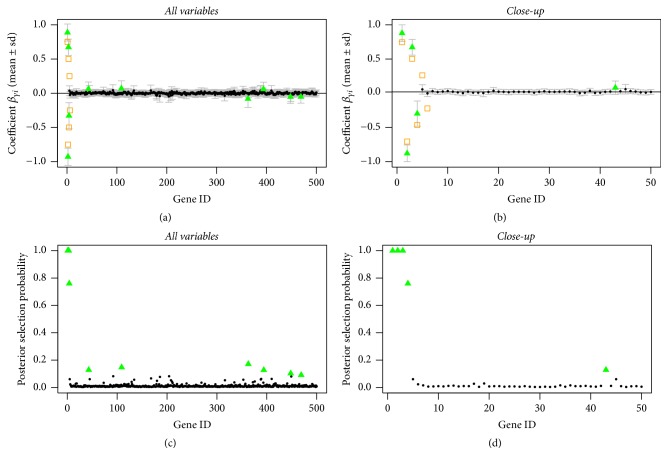
Sparse simulated data, uninformative prior: posterior mean estimates of *β* (a, b) and of selection probabilities (c, d) of all variables (a, c) and of the first 50 variables (b, d). Estimates are shown as black circles, selected variables as green triangles, and the true predictors as orange squares.

**Figure 2:**
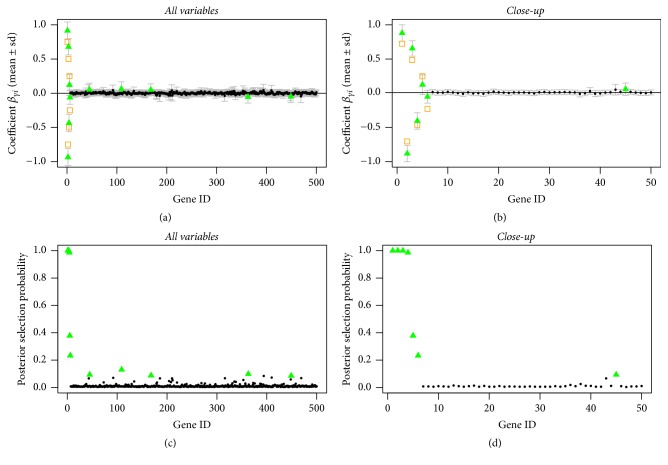
Sparse simulated data, correct informative prior: posterior mean estimates of *β* (a, b) and of selection probabilities (c, d) of all variables (a, c) and of the first 50 variables (b, d). Estimates are shown as black circles, selected variables as green triangles, and the true predictors as orange squares.

**Figure 3:**
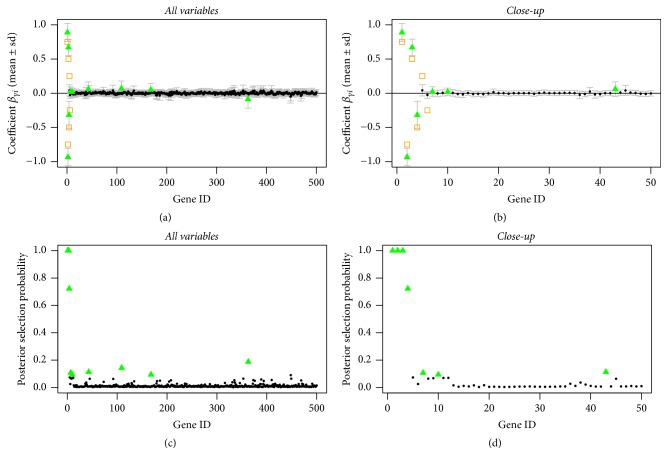
Sparse simulated data, incorrect informative prior: posterior mean estimates of *β* (a, b) and of selection probabilities (c, d) of all variables (a, c) and of the first 50 variables (b, d). Estimates are shown as black circles, selected variables as green triangles, and the true predictors as orange squares.

In Figures 1, 2, and 3, we can see that the true predictors with higher absolute effects of at least 0.5 are always selected, even for the setting where the prior probabilities are wrongly stated (compare Figure 3). The true predictors with smaller absolute effect sizes are less often selected, which is not surprising since with smaller underlying absolute effect sizes the posterior evidence of being one of the predictors is getting weaker.

This shows that in general the model is very robust with regard to wrongly stated prior information (Figure 3) or in the absence of information (Figure 1). The rate of wrongly selected variables does not differ much. However, when having prior information that comes close to the truth, even the variables with the smaller absolute effect sizes of 0.25 can be selected by the model, though their posterior selection probability is smaller than one; see Figure 2.

**Figure 4:**
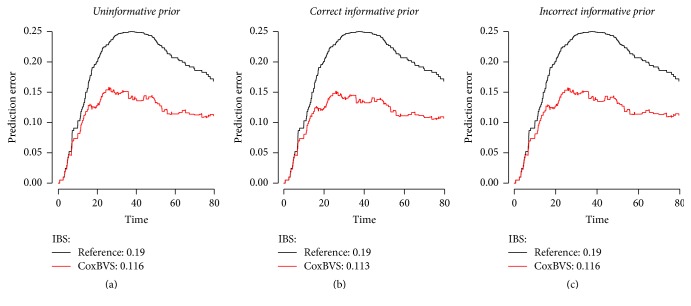
Prediction error curves of the simulated sparse test data up to time unit 80, based on the model containing only the selected variables with the largest posterior inclusion probabilities.

This is also confirmed by the prediction error curves and the IBS obtained for the test dataset in Figure 4. The difference in the prediction error curves between settings is not very big, since the identification of the effects is quite distinct in the sparse setting. The area between the curves and the integrated Brier score are the same with IBS = 0.16 for the uninformative (a) and incorrect (c) prior and slightly better for the correct informative prior with an IBS of 0.13 (b).

For the sparse setting, the mixing (i.e., the ability of the Gibbs sampler to move around in the model space) is very good and therefore the results are robust and consistent for the different scenarios (see Figures 12–15; the results of the sparse setting are shown in (a, b) of the figures). Because of the good initial mixing performance of the single Markov chains, the incorporation of parallel tempering does not further improve the mixing performance. Therefore, we only show the results for the single chain setups. For the parallel tempering, we obtained an acceptance rate of around 50% for swapped states of the Markov chains.

The MCMC mixing and convergence performances for the implementation with and without parallel tempering are illustrated in Figures 12–15.  Figure 12 shows running mean plots that illustrate the development of the posterior mean estimates of the regression coefficients *β* with increasing number of MCMC iterations. This shows how the estimates stabilize, thereby helping us to assess whether the MCMC sampler has run long enough. The running mean plots for the sparse simulation scenario indicate that the running means of *β* do not change much after ca. 10000 MCMC iterations.  Figure 13, which shows trace plots for the log likelihood functions, and Figures 14 and 15, which show trace plots for the regression coefficients *β*, are useful for deciding if the Markov chains are mixing well enough and to see if the MCMC sampler gets stuck in local optima. In addition, they can help with the decision for how long the burn-in period should be, that is, how many MCMC iterations at the start of the sampling process cannot be used for posterior estimation, because the sampler has not yet converged to the target distribution. All trace plots indicate very good mixing and show that the Markov chains move very fast (in less than 5000 MCMC iterations) to the best-performing model regions.

#### 3.1.2. Nonsparse Setting

**Figure 5:**
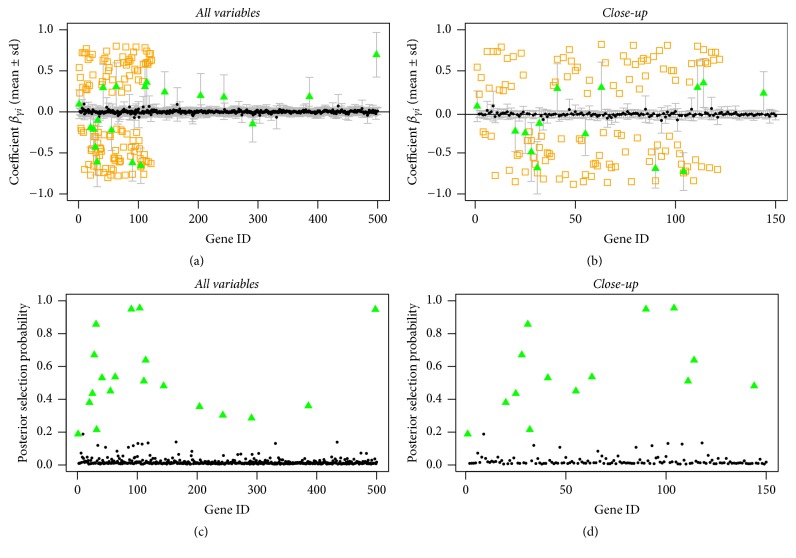
Nonsparse simulated data, uninformative prior: posterior mean estimates of *β* (a, b) and of selection probabilities (c, d) of all variables (a, c) and of the first 150 variables (b, d). Estimates are shown as black circles, selected variables as green triangles, and the true predictors as orange squares.

**Figure 6:**
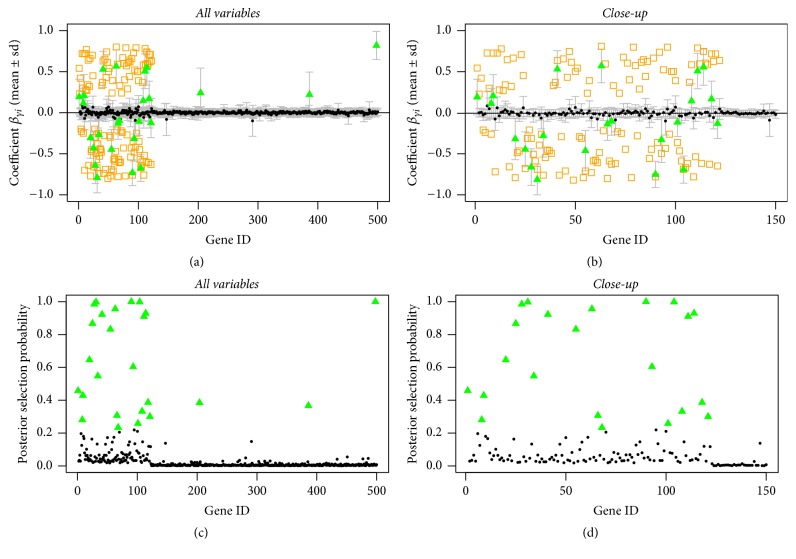
Nonsparse simulated data, correct informative prior: posterior mean estimates of *β* (a, b) and of selection probabilities (c, d) of all variables (a, c) and of the first 150 variables (b, d). Estimates are shown as black circles, selected variables as green triangles, and the true predictors as orange squares.

**Figure 7:**
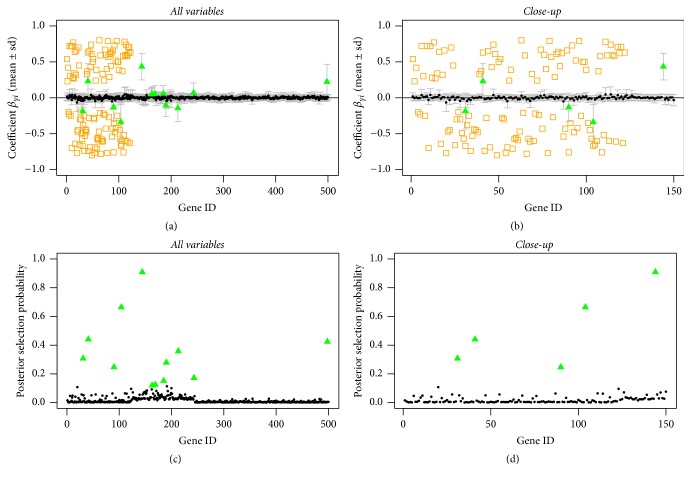
Nonsparse simulated data, incorrect informative prior: posterior mean estimates of *β* (a, b) and of selection probabilities (c, d) of all variables (a, c) and of the first 150 variables (b, d). Estimates are shown as black circles, selected variables as green triangles, and the true predictors as orange squares.

As a second evaluation step, we constructed a nonsparse scenario, where we generated *k*_true_ = 122 true predictors, again corresponding to the first *k* variables in the simulation setting. As expected in this case, the results are more inconsistent. In the nonsparse setting, the influence of the prior probabilities can be seen very nicely in the posterior selection probabilities (Figures 5, 6, and 7 (c, d), resp.). Variables with higher prior probability show a slight increase in the posterior selection rate. For the case with correctly specified informative prior probabilities, it can be seen that more of the true predictors are selected and the increase is more obvious than in the other cases (see Table 2). Furthermore, fewer of the nonpredictors are selected. When incorrect information is used to specify the prior probabilities (Figure 7), fewer of the true predictors will be selected as well as more of the false ones that obtained higher probability mass in the beginning. In the uninformative prior setting the model selects about 11% of the true predictors. With the correct informative prior 18% of the true predictors are selected and with incorrect informative priors we only identify 3% correctly (see Table 2). The posterior selection probabilities are shown in Figures 5, 6, and 7, where there is a clearer increase in the selection probabilities for the true predictors and generally smaller probabilities for the remaining nonpredictor variables (Figure 6).

**Figure 8:**
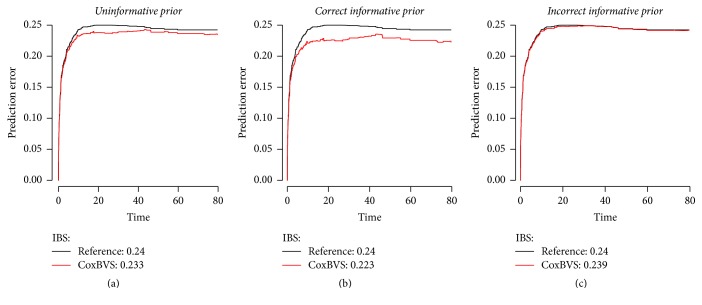
Prediction error curves of the simulated nonsparse test data up to time unit 80, based on the model containing only the selected variables with the largest posterior inclusion probabilities.

Additionally, we can see the impact of prior information more clearly from the prediction error curves obtained for the test data (Figure 8) where the prediction error is lowest for the correct informative prior with an IBS of 0.223 (a) compared to an IBS of 0.233 (b) for the uninformative prior and 0.239 (c) for the incorrect informative prior information case.

Again, we compared the results for the MCMC samplers with and without parallel tempering (see (c, d) of Figures 12–15). Since the nonsparse simulation scenario is more complex than the sparse scenario, we anticipated that the simple MCMC sampler (without parallel tempering) might need more iterations to move into the regions of the model space with the best-performing models or that the sampler might have problems with poor mixing. Indeed, we observe somewhat slower convergence (up to ca. 5000 MCMC iterations according to the trace plots in Figures 13–15). Therefore, parallel tempering can potentially be more useful in the nonsparse simulation scenario. However, we find that parallel tempering does not improve the mixing performance of the Markov chains sufficiently to justify the increase in computation time.

### 3.2. Glioblastoma

**Figure 9:**
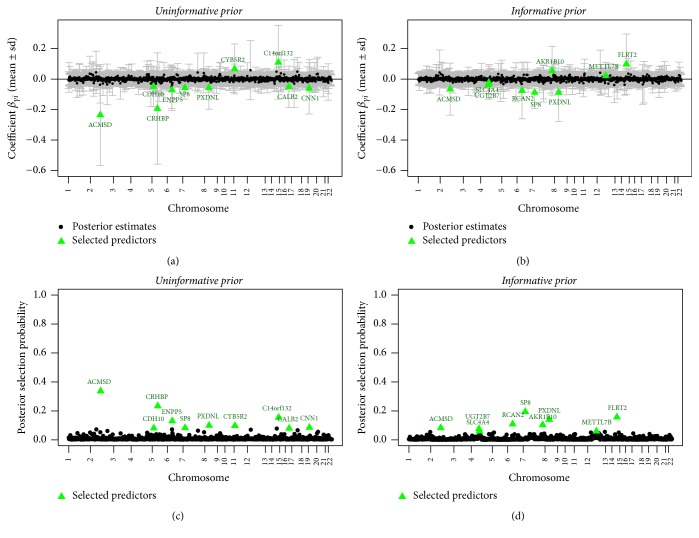
Glioblastoma data: posterior mean estimates or *β* (a, b) and of selection probabilities (c, d) for the uninformative (a, c) and informative (b, d) priors. The *p*_*m*_ = 10 (uninformative prior) and *p*_*m*_ = 9 (informative prior) selected variables are highlighted as green triangles, while estimates are shown as black circles.

Figure 9 summarizes the posterior estimates for *β* and *γ* for the glioblastoma application. Again, parallel tempering did not improve the Markov chain mixing sufficiently to outweigh the increased computational burden. Therefore, we performed the full MCMC runs only without parallel tempering.

**Figure 10:**
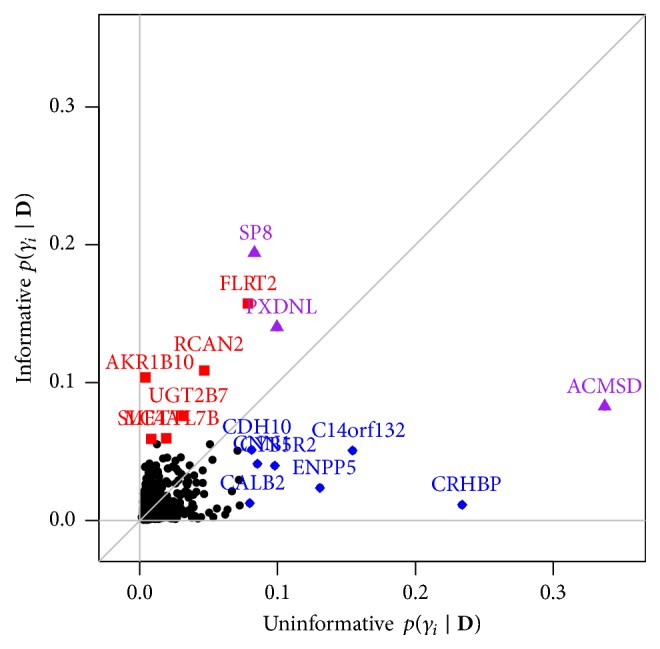
Glioblastoma data: posterior selection probabilities for the models based on the uninformative (*x*-axis) versus the informative (*y*-axis) priors. The highlighted data points correspond to the selected variables with the largest posterior selection probabilities based on the two priors (blue diamonds identify variables selected only with the uninformative prior, red squares only with the informative prior, and purple triangles with both priors; see Figure 9).

The posterior selection probabilities are quite different for the models with the informative and uninformative selection priors, respectively, as only 3 variables among the *p*_*m*_ variables with the largest marginal posterior selection probabilities for both priors; see also Figure 10. These are the genes with gene symbols ACMSD (on chromosome 2), SP8 (chromosome 7), and PXDNL (chromosome 8).

On average, across all MCMC iterations, the models contained *p*_*m*_ = 10 variables (uninformative prior) and *p*_*m*_ = 9 variables (informative prior), respectively. Therefore, for our top models, we select *p*_*m*_ variables with the largest posterior selection probabilities. The corresponding variables are highlighted in Figure 9 and their gene names are shown.  Table 3 gives an overview over the top genes including the gene symbols, full names, and the posterior selection probabilities.

ACMSD can prevent the accumulation of the neuronal excitotoxin quinolinate, which has been implicated in the pathogenesis of several neurodegenerative disorders (https://www.ncbi.nlm.nih.gov/gene/130013, updated 19-Jan-2017). This agrees with our finding of a negative regression coefficient estimate for ACMSD, since negative coefficients indicate a reduction in the hazard rate with an increase in gene expression. Not much is known about the roles of SP8 (https://www.ncbi.nlm.nih.gov/gene/221833, updated 6-Dec-2016) and PXDNL (https://www.ncbi.nlm.nih.gov/gene/137902, updated 6-Dec-2016) in human cancers or neurological diseases, but genetic variants in SP8 have been associated with psychotic disorders in recent genome-wide association studies in Han Chinese and Japanese populations [35, 36]. While some of the remaining genes are involved in neurological processes or neural development (CALB2, CDH10, ENPP5, and FLRT2), others have been associated with cancer (AKR1B10, CALB2, CDH10, and CYB5R2), but only CYB5R2 has specifically been identified as a potential (epigenetic) marker for glioblastoma prognosis [37].

**Figure 11:**
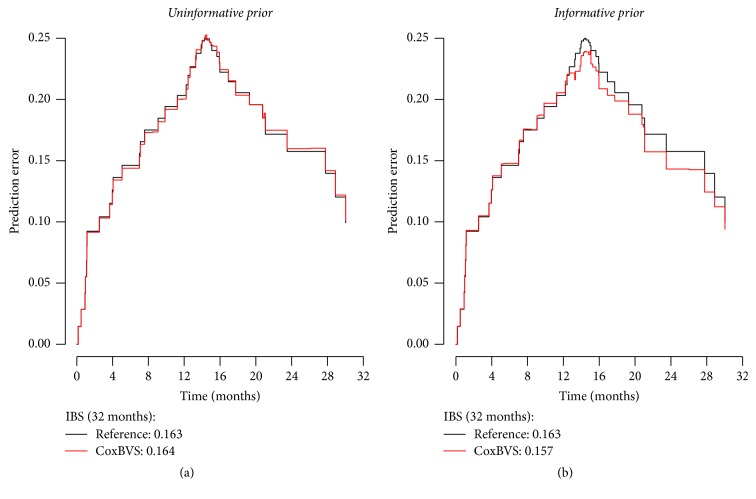
Glioblastoma data: prediction error curves for the test data based on the model containing only the selected variables with the largest posterior inclusion probabilities (see Figure 9).

**Figure 12:**
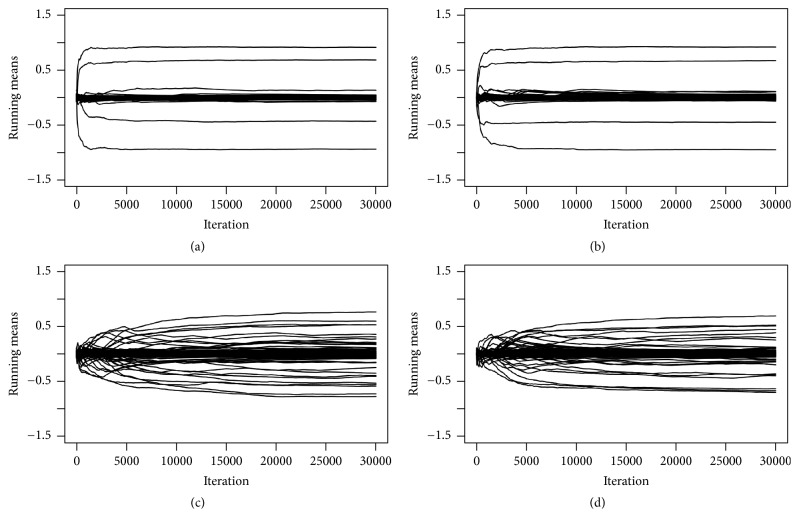
Simulation study: running mean plots of posterior for *β* for correct informative prior: sparse setting without (a) and with (b) parallel tempering and nonsparse setting without (c) and with (d) parallel tempering. For comparison purposes only, the first 30,000 iterations are shown.

**Figure 13:**
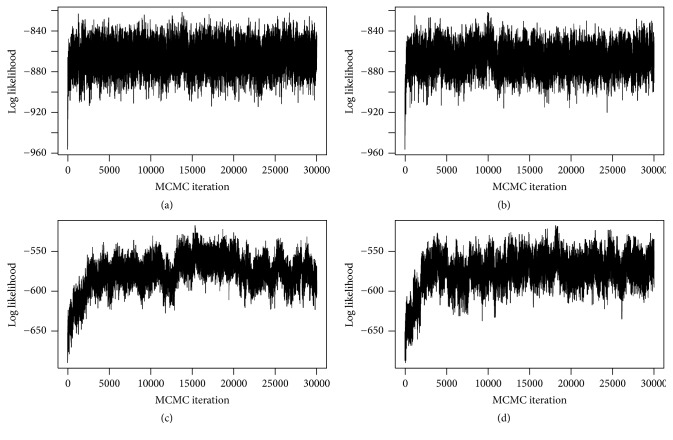
Simulation study: trace plots of log likelihood values for correct informative prior: sparse setting without (a) and with (b) parallel tempering and nonsparse setting without (c) and with (d) parallel tempering. For comparison purposes only, the first 30,000 iterations are shown.

**Figure 14:**
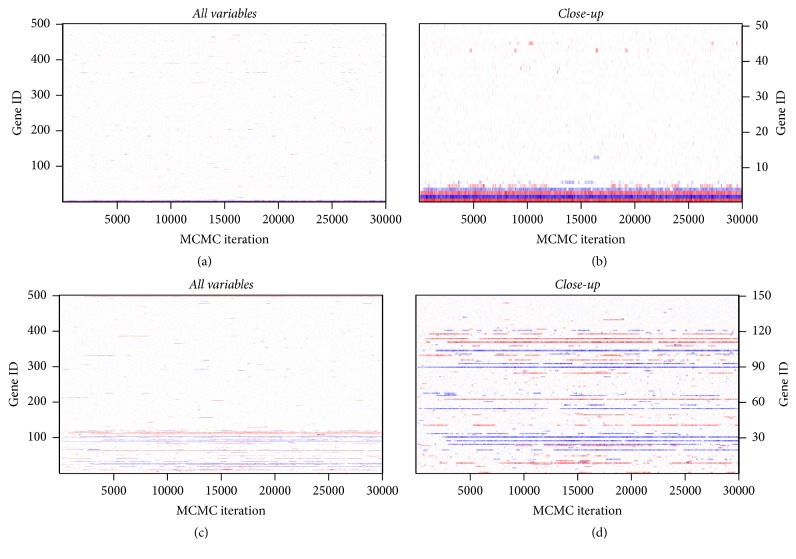
Simulation study: trace plots of *β* for correct informative prior without parallel tempering: sparse setting (a, b) and nonsparse setting (c, d) with all variables (a, c) and with first 50 or 150 variables (b, d). For comparison purposes only, the first 30,000 iterations are shown.

**Figure 15:**
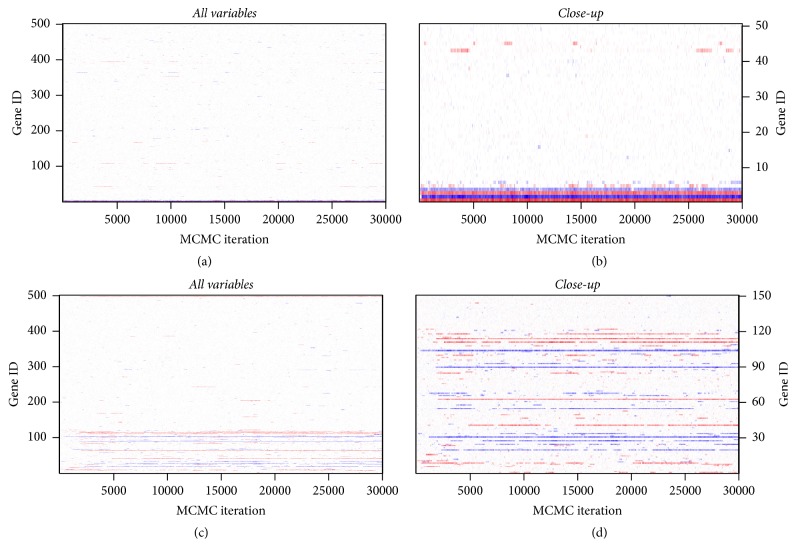
Simulation study: trace plots of *β* for correct informative prior with parallel tempering: sparse setting (a, b) and nonsparse setting (c, d) with all variables (a, c) and with first 50 or 150 variables (b, d). For comparison purposes only, the first 30,000 iterations are shown.

The prediction performance of the top models is evaluated in terms of the prediction error curves and integrated Brier scores (IBS) on the test dataset; see Figure 11. While the IBS for the model with the uninformative selection prior is not better than the IBS of the reference model (IBS = 0.163), we see a good improvement in the prediction performance for the model with the informative selection prior (IBS = 0.157), and the (test set) prediction error curve for the informative selection prior is lower than the reference prediction error curve, in particular after ca 12 months.

**Figure 16:**
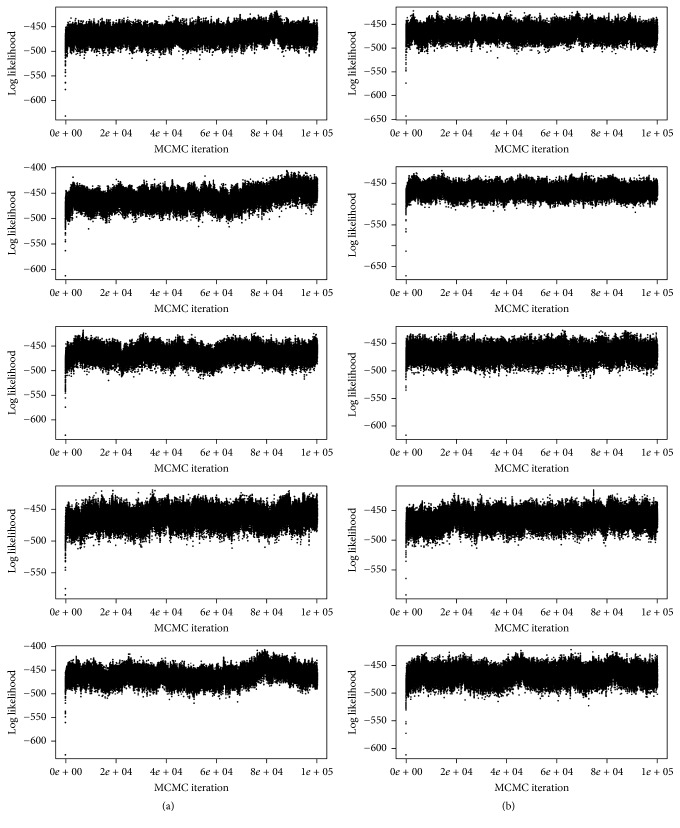
Glioblastoma data: trace plots of log likelihood values for all five MCMC chains for the uninformative prior (a) and for the informative prior (b).

For sampling diagnostics, we refer to Figure 16. It shows the trace plots for the log likelihood functions for all five MCMC chains that were run for sampling from the model with the uninformative selection prior (a) and correspondingly all five MCMC chains used for the informative selection prior (b). The trace plots demonstrate that all Markov chains move very fast (within the first 1000 MCMC iterations) to a region of the model space, where most model log likelihood values are in the range between ca. −500 and −450. The trace plots also show that the Markov chains do not get stuck in model regions with very similar log likelihood values, which indicates a good mixing performance.

## 4. Conclusion

In this manuscript, we have combined a Bayesian Cox model for survival data (Lee et al., 2011 [5]) with a variable selection approach suitable for high-dimensional input data (George and McCulloch, 1993 [10]). This approach of framing variable selection via Gibbs sampling over the binary indicator vector *γ* = (*γ*_1_,…, *γ*_*p*_) gave us the opportunity to integrate information from a second data source into the model via the prior distribution for *γ*. In our application to glioblastoma data, we integrated copy number variation data into a gene expression-based model for overall survival prognosis, and we found that the inclusion of the copy number data results in a better prediction performance in the test dataset.

This confirms our findings from the simulation studies that our model setup is able to use the second data source to achieve clear improvements in the prediction accuracy, if the second data source truly supplies an informative selection prior, that is, if the variables that are assigned an increased prior selection probability due to information in the secondary data source really are associated (in the main data source) with the response. An incorrect specification of the selection prior, however, might lead to slightly worse prediction performance compared to the uninformative selection prior. In real applications, we will typically not know if an informative selection prior is specified correctly. Therefore, it is important to always compare the prediction performance of such an informative prior with the uninformative (standard) prior to see whether or not prediction performance is improved by the prior information. In general, a sensitivity analysis to assess the impact of the choice of priors on the results is a recommended procedure for any Bayesian analysis, especially when using informative priors.

The advantage of our fully Bayesian modeling approach compared to frequentist approaches is that we obtain full inference, not only for the posterior distributions of the regression coefficients *β*, but also for the posterior selection probabilities of all the variables. Note that due to the joint modeling we can even obtain posterior inference about the joint selection probabilities of specific sets of variables. In this way, we can explore how the selection of one variable affects the selection probability of another variable, or we can estimate and compare the joint posterior selection probabilities of specific (published) gene signatures, that is, sets of genes that have been identified as being prognostic in previous studies. Since we essentially use the Gibbs sampler to perform a stochastic search over the model space of size 2^*p*^ (with *p* easily being in the hundreds or thousands), it is not feasible to run the MCMC sampler long enough for reliable posterior estimation in the low-probability regions. However, this is usually not a concern, since we are mostly interested in the variables and models with highest posterior selection probabilities. Because of the nature of the stochastic search sampler to visit models with a frequency that is proportional to their posterior selection probability, it is much easier to obtain a sufficient number of MCMC samples for good estimation performance for these high-probability models.

In general, there is a trade-off between the computational expense of longer MCMC runs and the improvement in estimation accuracy, both by reducing the MCMC error and by ensuring that the relevant high-probability model regions have been visited with sufficient frequency. Increasing the number of variables *p* that are considered in the modeling process will also increase the computational expense. Here a good trade-off is achieved if the number of variables without predictive value with regard to the survival outcome is kept to a minimum. Our implementation of the algorithm in R has not been optimized with respect to computing performance and the computing speed could be improved substantially, for example, by using the R package Rcpp [38] and by more efficient memory management. Currently, a single MCMC run in our simulation studies and data application takes ca. one hour per 1000 MCMC iterations on a 2.6 GHz compute node running Linux with 64 GB memory; all results presented in this manuscript are based on MCMC runs that took a maximum of one week running time.

We found in our applications that the parallel tempering algorithm did not sufficiently improve the mixing performance of the Markov chains (i.e., the ability of the Gibbs sampler to move around in the space of all models) to offset the increase in computation time. The increase in computation time can be minimized by implementing the parallel tempering with true computational parallelization, for example, by running each of the tempered Markov chains on a different node. In that case, the only increase in computation time comes from the necessary regular exchanges of the states of the Markov chains between neighboring tempered chains. Thus, parallel tempering might be much more favorable in such an implementation. However, note that another trade-off is involved, namely, the increase in computation time and the improvement in mixing performance due to an increased frequency of state exchanges. See [39] for a simple example implementation in R, which illustrates the procedure.

## Figures and Tables

**Table 1:** Simulation study: posterior selection probabilities for *k*_true_ = 6 true variables of the sparse setting.

Prior setting	Variable index					
	1	2	3	4	5	6
Uninformative	1.000	1.000	1.000	0.760	0.061	0.023
Correct informative	1.000	1.000	1.000	0.986	0.379	0.233
Incorrect informative	1.000	1.000	1.000	0.722	0.073	0.026

**Table 2:** Simulation study (sparse and nonsparse scenario): variable selection results (selection of *p*_*m*_ variables, where *p*_*m*_ is the mean model size as described in Section 2.4).

Prior setting	Sparse setting				Nonsparse setting			
	#TP	#FN	#FP	#TN	#TP	#FN	#FP	#TN
Uninformative	4	2	6	488	13	109	6	372
Correct informative	6	0	5	489	22	100	3	375
Incorrect informative	4	2	6	488	4	118	8	370

#TP = number of true positives, #FN = number of false negatives, #FP = number of false positives, and #TN = number of true negatives.

**Table 3:** Glioblastoma data: overview of the genes among *p*_*m*_ variables with the largest marginal posterior selection probabilities in the model with either the uninformative or the informative prior.

Symbol	Full name	*p*_1_(*γ*_*i*_ | **D**)^*∗*^	*p*_2_(*γ*_*i*_ | **D**)^+^
ACSMD	Aminocarboxymuconate semialdehyde decarboxylase	0.337	0.083
AKR1B10	Aldo-keto reductase family 1 member B10		0.104
CALB2	Calbindin 2	0.080	
CDH10	Cadherin 10	0.081	
CNN1	Calponin 1	0.085	
CRHBP	Corticotropin releasing hormone binding protein	0.234	
CYB5R2	Cytochrome b5 reductase 2	0.098	
C14orf132	Chromosome 14 open reading frame 132	0.154	
ENPP5	Ectonucleotide pyrophosphatase/phosphodiesterase 5	0.131	
FLRT2	Fibronectin leucine rich transmembrane protein 2		0.157
METTL7B	Methyltransferase like 7B		0.060
PXDNL	Peroxidasin like	0.100	0.140
RCAN2	Regulator of calcineurin 2		0.109
SLC4A4	Solute carrier family 4 member 4		0.059
SP8	Sp8 transcription factor	0.083	0.194
UGT2B7	UDP glucuronosyltransferase family 2 member B7		0.076

^*∗*^ *p*_1_(*γ*_*i*_ | **D**) denotes the posterior selection probability in the model with the uninformative selection prior and ^+^*p*_2_(*γ*_*i*_ | **D**) denotes the posterior selection probability in the model with the informative selection prior.
